# The influence of CD26^+^ and CD26^−^ fibroblasts on the regeneration of human dermo-epidermal skin substitutes

**DOI:** 10.1038/s41598-022-05309-5

**Published:** 2022-02-04

**Authors:** Katarzyna Michalak-Micka, Agnes S. Klar, Athanasia Dasargyri, Thomas Biedermann, Ernst Reichmann, Ueli Moehrlen

**Affiliations:** 1 Tissue Biology Research Unit, Department of Surgery, University Children’s Hospital Zurich, University of Zurich, Steinwiesstrasse 75, 8032 Zurich, Switzerland; 2 Children’s Research Center, University Children’s Hospital Zurich, Zurich, Switzerland; 3 Department of Surgery, University Children’s Hospital Zurich, University of Zurich, Zurich, Switzerland; 4 University of Zurich, Zurich, Switzerland

**Keywords:** Regenerative medicine, Tissue engineering

## Abstract

CD26, also known as dipeptidyl peptidase IV (DPPIV), is a multifunctional transmembrane protein playing a significant role in the cutaneous wound healing processes in the mouse skin. However, only scarce data are available regarding the distribution and function of this protein in the human skin. Therefore, the aim of this study was to investigate the impact of CD26 deficiency in human primary fibroblasts on the regeneration of human tissue-engineered skin substitutes in vivo. Dermo-epidermal skin analogs, based on collagen type I hydrogels, were populated either with human CD26^+^ or CD26^knockout^ fibroblasts and seeded with human epidermal keratinocytes. These skin substitutes were transplanted onto the back of immune-incompetent rodents. Three weeks post-transplantation, the grafts were excised and analyzed with respect to specific epidermal and dermal maturation markers. For the first time, we show here that the expression of CD26 protein in human dermis is age-dependent. Furthermore, we prove that CD26^+^ fibroblasts are more active in the production of extracellular matrix (ECM) both in vitro and in vivo and are necessary to achieve rapid epidermal and dermal homeostasis after transplantation.

## Introduction

Fibroblasts are the most prevalent cell type in the dermis. They are embryonically derived from the mesenchyme and synthetize and deposit certain components of the extracellular matrix (ECM)^1,2^. They play key roles not only in wound healing and scarring but also in inflammation^3^, angiogenesis^4,5^, as well as in progression and invasion of tumors^6^. In the past, fibroblasts had been considered as a homogenous population of dermal cells. However, recent studies have shed more light on the complexity and the diversity of fibroblasts in the dermis.

The dermis can be divided into two anatomically distinct layers. The papillary dermis is a superficial region located just underneath the dermo-epidermal junction. It is characterized by the presence of thin and poorly organized collagen type I bundles that provide structural support to the overlying epithelium. In contrast, the reticular dermis is a deeper skin layer composed of thick collagen type I bundles arranged together in a basketweave pattern^7^. Interestingly, papillary and reticular fibroblasts isolated and cultured from mechanically separated human dermis by a dermatome device exhibit differences in terms of proliferation, contraction as well as in gene expression pattern^8–11^. However, dermatome approaches, although very successful, are often limited due to the inability to precisely control the depth of sectioning. Therefore, the presence of reticular fibroblasts in expand cultures of papillary fibroblasts cannot be excluded.

More recent studies on mice models have revealed that papillary and reticular fibroblasts arise from one multipotent mesenchymal progenitor^12^. Interestingly, lineage commitment in mice occurs by embryonic day E16. Until postnatal day 2, DLK1 and CD26 cell surface markers distinguish between papillary and reticular fibroblasts. Thereafter, the expression of DLK1 gradually decreases, while CD26 remains to be expressed in reticular fibroblasts lineage (including derma papilla cells in telogen phase of hair follicle cycle)^13–15^. In addition, papillary fibroblasts are required for the formation and growth of new hair follicles while reticular fibroblasts secrete the ECM and contribute to the skin repair and regeneration after wounding^12^.

Several alternative approaches have been performed to further understand the heterogeneity of fibroblasts within the skin. Haydont et al. have conducted genome-wide molecular profiling of papillary and reticular fibroblasts isolated from human skin of young and old donors. Notably, the group has revealed that chronological ageing in the human skin leads to the significant modulations of transcripts in papillary and reticular fibroblasts. Those modifications are associated with the biology of ECM, cytoskeleton and focal adhesion points^16^.

Using lineage-tracing experiments, Rinkevich et al. identified Engriled-1 (EN1) positive fibroblasts, as the cell lineage responsible for the deposition of ECM in embryonic development, cutaneous wound healing as wells as in post-radiation fibrosis in a mouse model. In addition, the authors revealed that dipeptidyl peptidase IV (DPPIV also known as CD26) is a cell surface marker allowing the enrichment of EN1-positive, pro-fibrotic fibroblasts^17^. Interestingly, extension of these research efforts from mice to humans showed that DPPIV/CD26 is not only expressed in mice but also in human fibroblasts^18–20^. Single-cell RNA sequencing on cells isolated from whole human skin samples discriminated between two major fibroblasts subpopulations, one of which is characterized by the expression of FMO1/LSP1 and the second expressing DPPIV/CD26^19^. Of note, only this fibroblast lineage exhibited a high expression of collagen, fibrillin and fibronectin suggesting its significant role in ECM deposition^19^. However, the majority of research regarding the role of DPPIV/CD26 still originates from mouse studies. Reports describing the exact distribution and function of CD26 in the human skin are inconsistent and still require further investigation.

Therefore, in this study, we investigated the influence of CD26 in fibroblasts on human skin regeneration using our established animal model. We used human CD26^+^ and CD26^knockout^ fibroblasts in combination with human keratinocytes in an in vivo assay on immune-incompetent rats. Skin graft maturation and homeostasis were analyzed 3 weeks after transplantation. We observed that skin grafts containing CD26^+^ fibroblasts are faster in the achievement of skin homeostasis, as compared to CD26^knockout^ cells.

## Results

### The expression of CD26 protein in human fibroblasts increases with age

**Figure 1:**
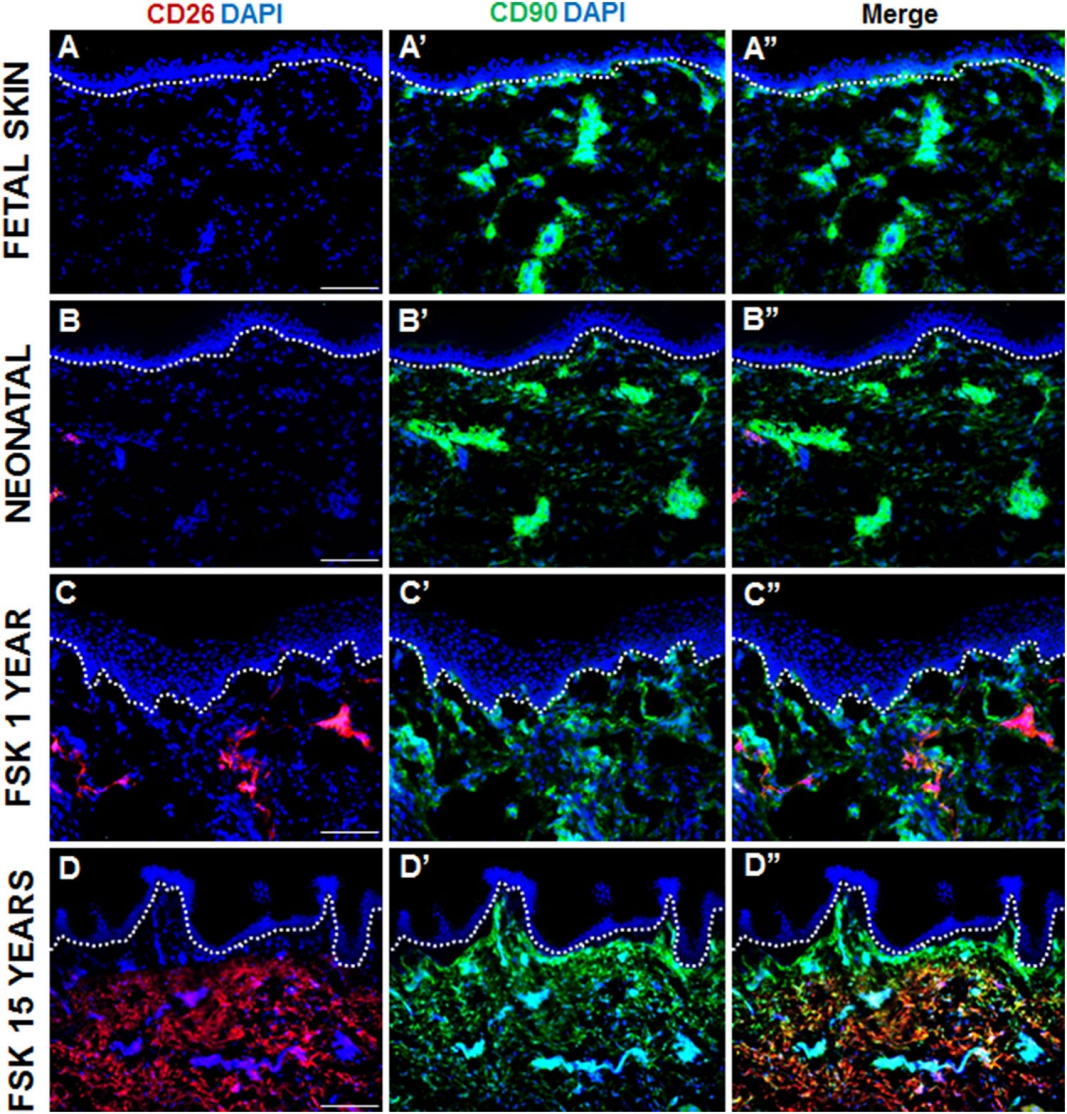
Expression pattern of CD26 and CD90 in human skin samples at different ages. (**A**–**B”**) Staining of CD26 (red) and CD90 (green) in (**A–A”**) fetal skin as well as in (**B**–**B”**) neonatal skin. Note that CD26 is expressed at the marginal level in both types of skin samples. (**C–D”**) Staining of CD26 (red) and CD90 (green) in foreskin from (**C–C”**) a 1 year old donor and (**D–D”**) a 15 years old donor. Note that in the foreskin form a 1 year old donor, CD26-expressing cells form clusters distributed randomly throughout the dermis. In the 15 years old donor, CD26 expression is restricted to reticular dermis. Dotted lines indicate dermo-epidermal junction. Blue indicates cell nuclei. Scale bar: 100 µm.

To examine in depth the expression of CD26 in human skin in situ, we performed combined immunofluorescence stainings for CD26 (red), and CD90 (green) delineating fibroblasts in the human dermis (Fig. 1A–D”). Interestingly, we observed that the expression of CD26 in human skin is strongly age-dependent. In fetal and neonatal dermis, the expression of this protein is marginal (Fig. 1A–A” and B–B”) and significantly increases with human age. In the foreskin sample from a 1 year old donor, CD26 expressing cells congregate forming specific cell clusters dispersed randomly throughout the dermis (Fig. 1C–C”). Interestingly, older donors show enhanced CD26 expression, which is localized exclusively in the reticular dermis (Fig. 1D–D”).

**Figure 2:**
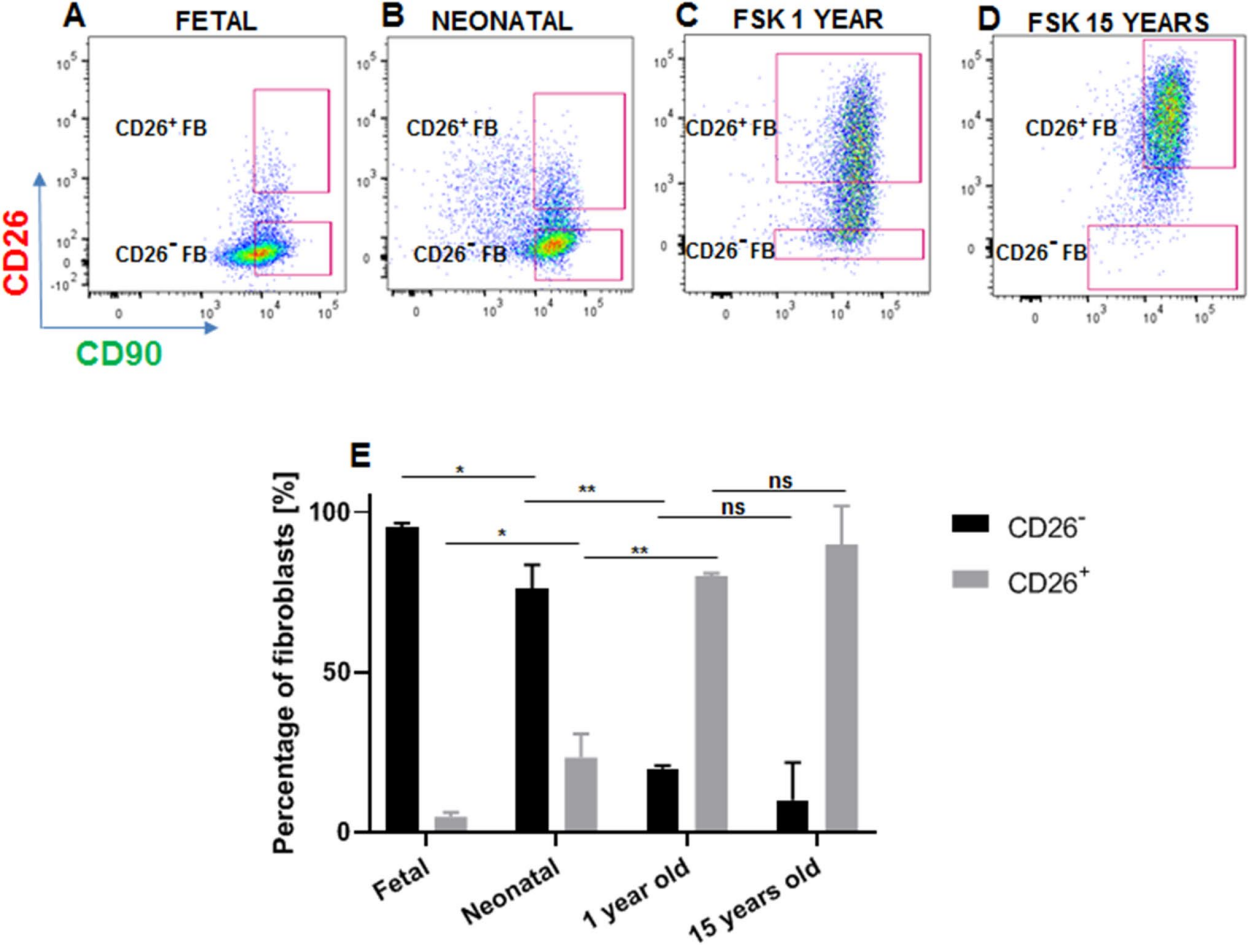
Flow cytometry analysis performed of human primary fibroblasts isolated from skin samples at different ages. (**A–D**) Flow cytometry analysis showing the abundance of CD26^−^ and CD26^+^ fibroblasts in (**A**) fetal skin, (**B**) neonatal skin, (**C**) foreskin from a 1 year old donor and (**D**) foreskin from a 15 years old donor. Note a shift in the population dynamics from CD26^–^dominated dermis in (**A**) fetal and (**B**) neonatal skin to the CD26^+^-dominated dermis in foreskin from (**C**) a 1 year old and (**D**) a 15 years old donor. (**E**) Graph showing the percentage of CD26^-^ and CD26^+^ fibroblasts in each skin sample. For each age, cells were analyzed from 3 independent skin samples. Results are presented as a mean ± SD. p-values were calculated using unpaired student t-test *indicates p-value 0.01–0.05 (significant); **indicates p-value 0.001–0.01 (very significant); ns indicates P-value not significant (p > 0.05). *FSK* foreskin.

To further investigate the age-related CD26 expression in human skin, we performed flow cytometry analysis on human primary fibroblasts isolated from donors at different ages (Fig. 2A–E). During FACS, the gating strategy involved the exclusion of doublets as well as dead cells. Isotype controls were used to identify the background signal and correctly set gates for positive cells (Suppl. Fig. 1A–D). In human fetal skin, the CD26^+^ fibroblast population represented 3.8 ± 1.3% of total dermal fibroblasts, which at this stage were dominated by CD26^−^ cells (95.2 ± 1.3%). With age, the CD26^+^ population increased from 23.3 ± 7.3% of total dermal fibroblasts in neonatal skin, to 80.1 ± 0.8% in 1 year old foreskin, and to 89.9 ± 12% in 15 years old foreskin. Subsequently, CD26^+^ and CD26^-^ fibroblast subpopulations from above mentioned skin samples were sorted using FACS and cultivated in vitro for further experiments.

### CD26^-^ fibroblasts regain the CD26 expression during in vitro cultivation

**Figure 3:**
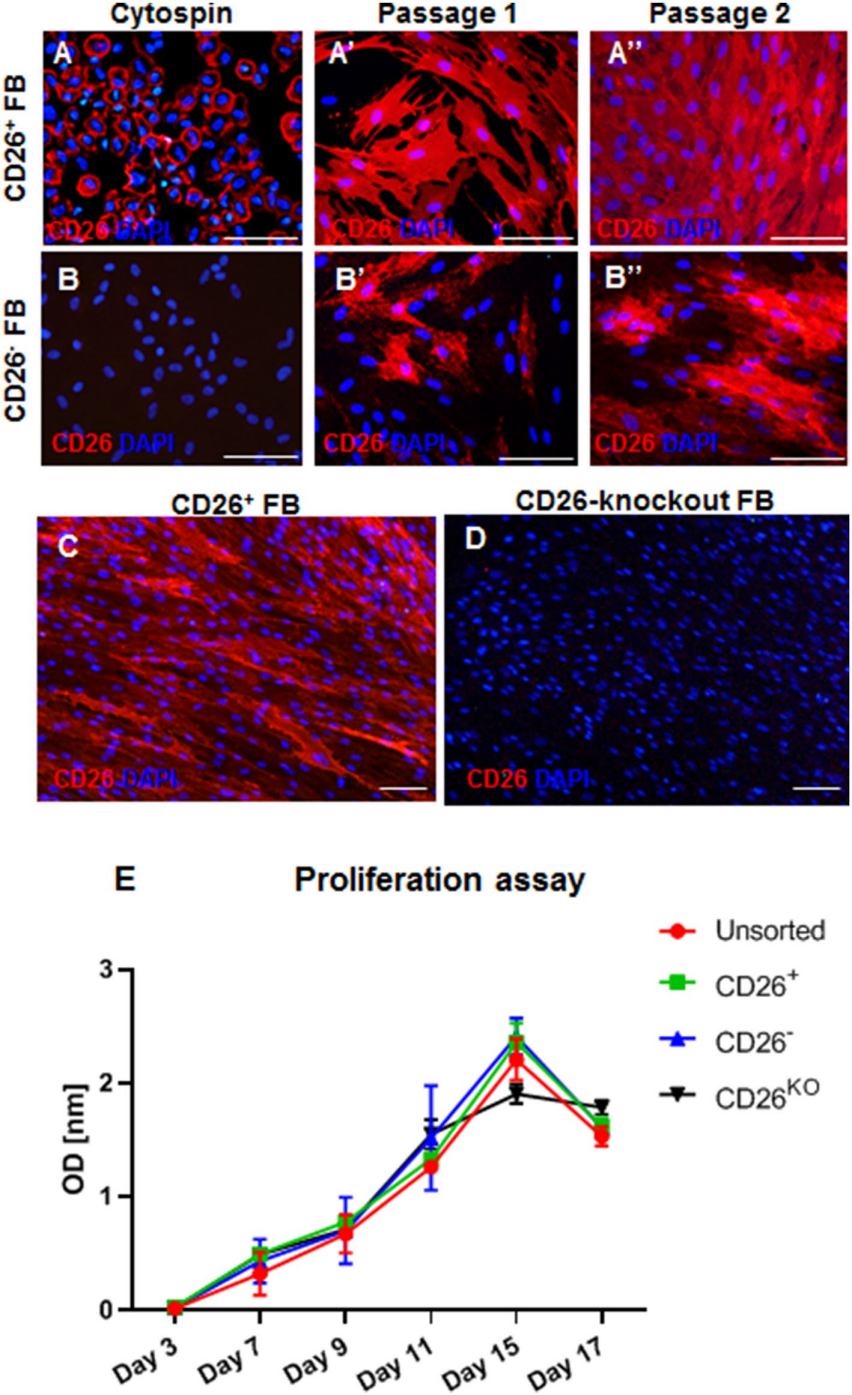
Expression of CD26 in sorted CD26^+^ and CD26^−^ fibroblasts and proliferation rates of CD26^+^, CD26^−^, CD26^knockout^ and unsorted fibroblasts. (**A**, **B”**) Representative immunofluorescence pictures showing the expression of CD26 in CD26^+^ and CD26^−^ fibroblasts (**A**–**B**) directly after sorting (cytospin), (**A’**, **B’**) at passage 1, and (**A”**, **B”**) at passage 2. Note that CD26^−^ fibroblasts gain the CD26 expression during in vitro cultivation. (**C**, **D**) Representative immunofluorescence pictures exhibiting the expression of CD26 in CD26^+^ and CD26^knockout^ fibroblasts. Note that CD26^knockout^ fibroblasts entirely lack the CD26 expression. Scale bars: 100 µm. (**E**) Colometric proliferation assay revealed the similar proliferation rates of CD26^+^, CD26^−^, CD26^knockout^ and unsorted fibroblasts. Results are presented as a mean ± SD (n = 3). p-values were calculated using unpaired student t-test. ns indicates p-value > 0.5 (not significant).

To investigate the stability of CD26 expression during in vitro cultivation, we immunostained CD26^+^ and CD26^−^ fibroblast subpopulations isolated from foreskin samples (1 and 15 years old donors) for CD26 directly after sorting (without further cultivation) as well as at different passages. We observed that sorted and directly cyto-spinned CD26^+^ fibroblasts exhibited a strong expression of CD26, while CD26^-^ cells were entirely negative for this marker (Fig. 3A,B). However, already at passage 1 (P1), we observed the presence of CD26-expressing cells in the CD26^-^ population (Fig. 3A’,B’). Of note, at higher passages, the CD26^-^ fibroblast subpopulation was almost utterly dominated by CD26-positive cells (Fig. 3A”,B”).

Given that CD26^-^ fibroblasts are likely to gain the expression of CD26 protein in vitro, we performed a CD26 knockout on human primary fibroblasts using lentiviral vectors for CRISPR/Cas9 genome editing. Successful knockout of the gene of interest was verified by CD26 staining. As depicted in Fig. 3C,D, CD26^knockout^ fibroblasts entirely lack the expression of CD26, whereas it was positive in the CD26^+^ population (Fig. 3C,D).

The proliferation rate of CD26^+^, CD26^-^, CD26^knockout^ and unfractioned fibroblasts was assayed for 17 days in vitro (Fig. 3E). We observed that all four fibroblasts subgroups exhibit similar proliferation ability peaking at day 15 (CD26^+^ 2.3 ± 0.2; CD26^−^ 2.4 ± 0.2; CD26^knockout^ 1.9 ± 0.09; unsorted 2.2 ± 0.2).

### CD26^+^ fibroblasts exhibit pro-fibrotic phenotype in vitro

To further characterize the phenotype of sorted CD26^+^ and CD26^−^ fibroblast subpopulations, we performed immunofluorescence stainings using typical pro-fibrotic markers.

**Figure 4:**
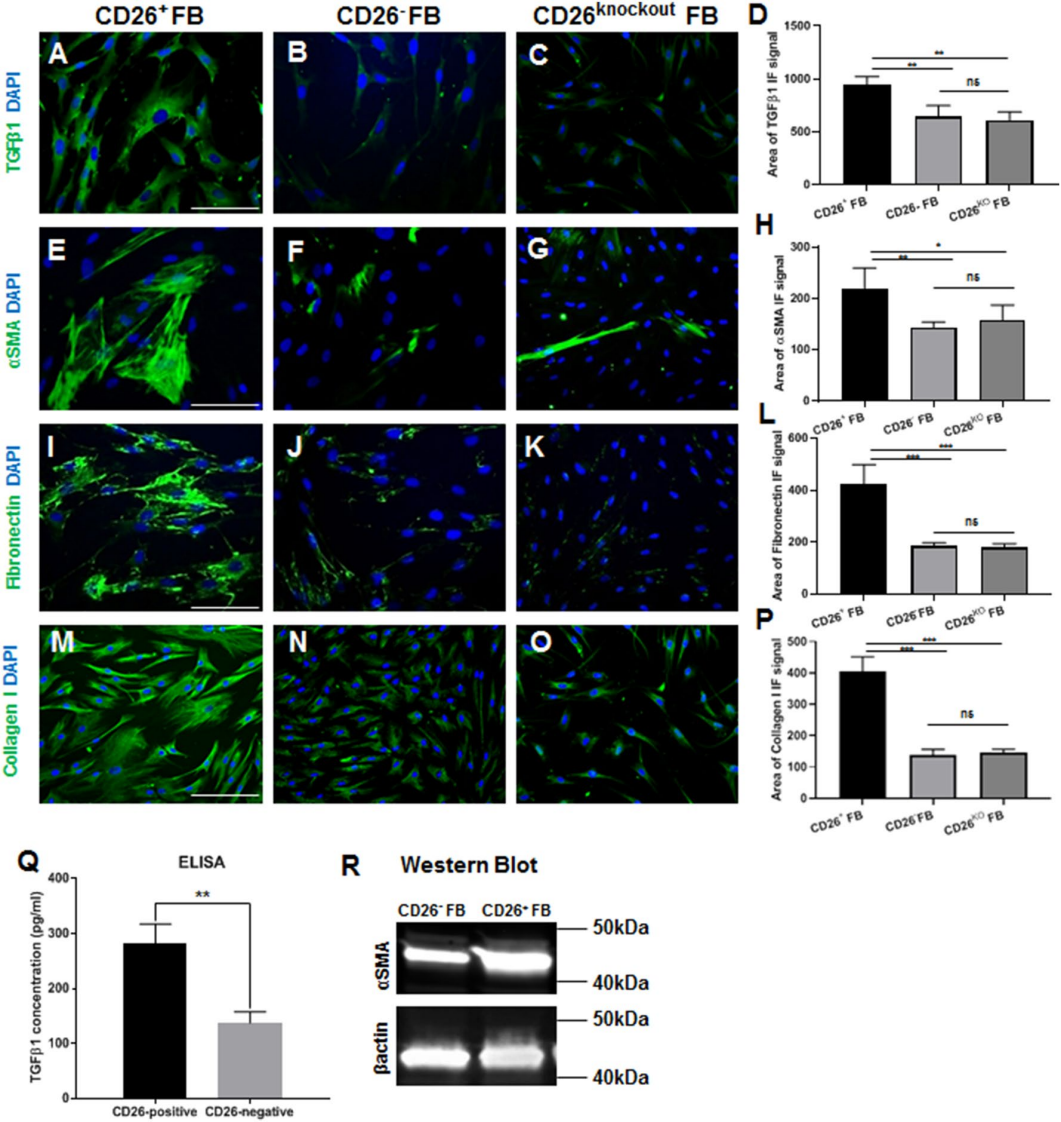
Characterization of CD26^+^, CD26^−^ and CD26^knockout^ fibroblasts in 2D culture in vitro. (**A**–**P**) Representative immunofluorescence pictures showing the expression of (**A**–**D**) TGFβ1, (**E**–**H**) αSMA, (**I**–**L**) fibronectin and (**M**–**P**) collagen I (all in green) in sorted and cultured CD26^+^, CD26^-^ and CD26^knockout^ fibroblasts. Please note that the quantification of immunofluorescence signal revealed that CD26^+^ cells express significantly more TGFβ1, αSMA, fibronectin and collagen I, as compared to the CD26^-^ and CD26^knockout^ fibroblasts. Results are presented as a mean ± SD (n = 5). p-values were calculated using unpaired student t-test. *Indicates p-value 0.01 to 0.05 (significant); **indicates p-value 0.001–0.01 (very significant); ***indicates p-value p < 0.001 (extremely significant); ns indicates P-value not significant. (**Q**) ELISA of TGFβ1 protein isolated from supernatants of cultured dermal CD26^+^ and CD26^−^ fibroblasts. Results are presented as a mean ± SD (n = 5). p-values were calculated using unpaired student t-test. **Indicates p-value 0.001–0.01 (very significant). (**R**) Western blot analysis of αSMA protein isolated from cell lysates of cultured dermal CD26^+^ and CD26^−^ fibroblasts. Blue indicates cell nuclei. Scale bar: 100 µm. *TGFβ1* transforming growth factor-β1, *αSMA* αSmooth Muscle Actin, *ELISA* enzyme-linked immunosorbent assay.

For the comparisons we used fibroblasts isolated from foreskin samples (1 and 15 years old donors). We found that in 2D cultures, CD26^+^ fibroblasts express significantly more transforming growth factor β-1 (TGFβ-1) (Fig. 4A,B), α-smooth muscle actin (α-SMA) (Fig. 4E,F), fibronectin (Fig. 4I,J) as well as collagen I (Fig. 4M,N), in comparison to CD26^-^ cells. In addition, western blot revealed a higher expression of αSMA in CD26^+^ fibroblasts as compared to CD26^−^ population, confirming our immunofluorescence data (Fig. 4R, raw data in Suppl. Fig. 2).

To assess the concentrations of TGFβ-1 in conditioned media (CM) of both fibroblast subpopulations, an enzyme-linked immunosorbent assay (ELISA) was performed (Fig. 4Q). As expected, TGFβ-1 concentrations in CD26^+^-CM were twofold higher than in CD26^–^CM (p = 0.0036).

In addition, we have performed immunofluorescence staining to assess whether the CD26 knockout affects the expression of pro-fibrotic markers. Quantification of the immunofluorescence signals revealed that similarly to CD26^-^ fibroblasts, CD26^knockout^ cells express low levels of TGFβ-1 (Fig. 4C,D), αSMA (Fig. 4G,H), fibronectin (Fig. 4K,L) as well as collagen type I (Fig. 4O-P). We would like to highlight that there was no significant difference in the expression of respective markers between CD26^-^ and CD26^knockout^ fibroblasts.

### The expression of CD26 and CD90 in transplanted human skin analogs

**Figure 5:**
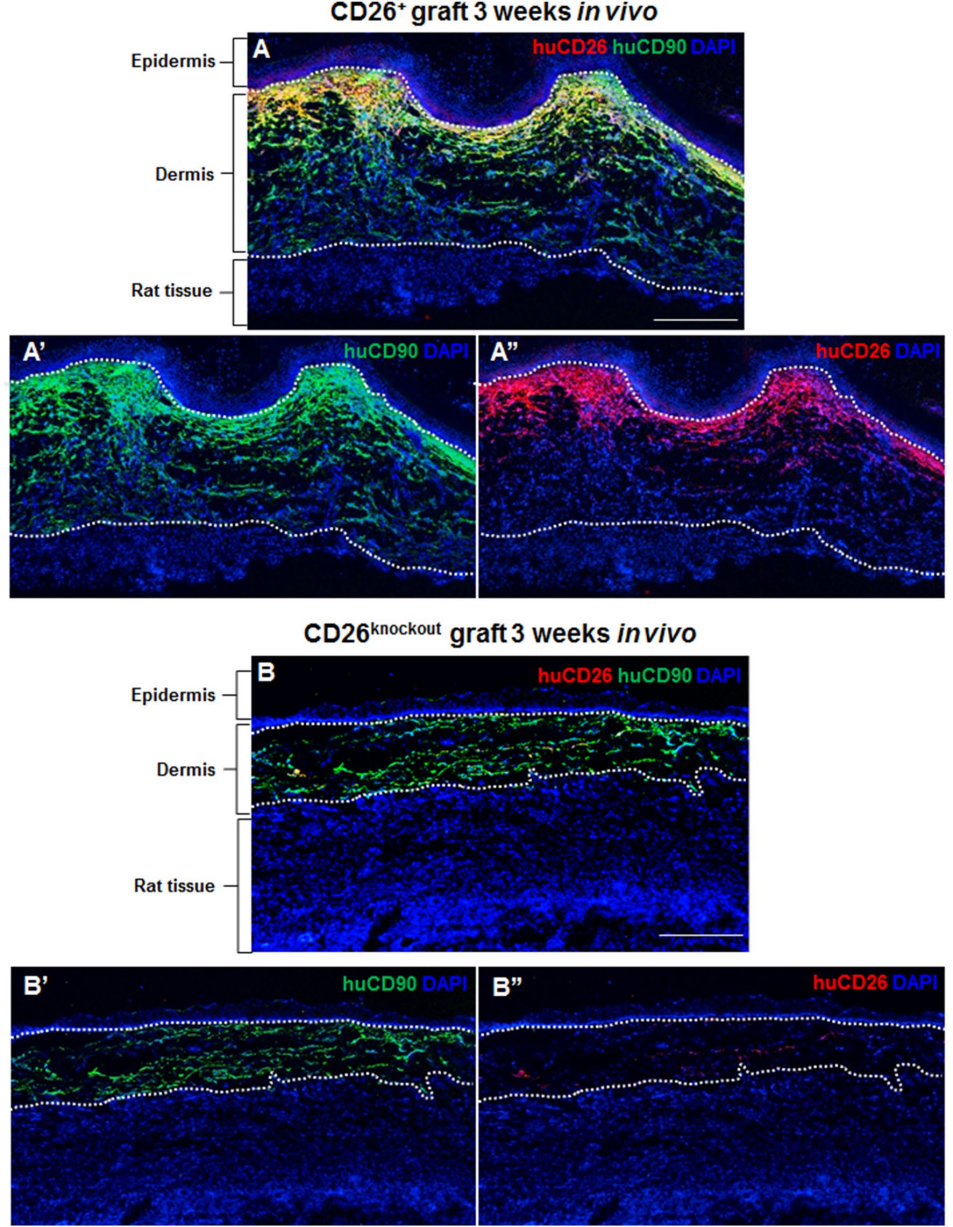
Expression pattern of CD26 and CD90 human dermo-epidermal skin substitutes 3 weeks after transplantation. (**A**–**B”**) Immunofluorescence staining of CD26 (red) and CD90 (green) in skin grafts containing (**A**–**A”**) CD26^+^ and (**B**–**B”**) CD26^knockout^ fibroblasts. Human CD90 positive fibroblasts (green) are localized in the dermal compartments of both (**A**) CD26^+^ and (**B**) CD26^knockout^ skin grafts underneath a stratified, multilayered epithelium and above the rat mesenchymal cells. Note the strong dermal expression of CD26 in skin analogs with CD26^+^ fibroblasts and almost no expression of this marker in skin grafts with CD26^knockout^ cells. Dotted lines indicate the dermo-epidermal junction and the border between rat and human tissue. Blue indicates cell nuclei. Scale bar: 500 µm.

To investigate the effect of CD26 in fibroblasts on human skin regeneration, collagen type I hydrogels containing either CD26^+^ or CD26^knockout^ fibroblasts were covered with human foreskin keratinocytes and subsequently transplanted onto the back of immune-incompetent rats. After 3 weeks in vivo, the presence of human fibroblasts in the neodermis was assessed using a CD90 antibody which detects only fibroblasts of human origin but not host (rat) dermal cells (Fig. 5A–B”). We found that in both types of transplants human fibroblasts were localized underneath the multilayered, stratified epithelium. Furthermore, we detected a strong expression of CD26 in skin grafts containing CD26^+^ fibroblasts (Fig. 5A–A”) and almost no visible expression of this cell surface marker in skin composites composed of CD26^knockout^ cells (Fig. 5B–B”).

### The impact of CD26^+^ and CD26^knockout^ fibroblasts on the epidermal regeneration in vivo

**Figure 6:**
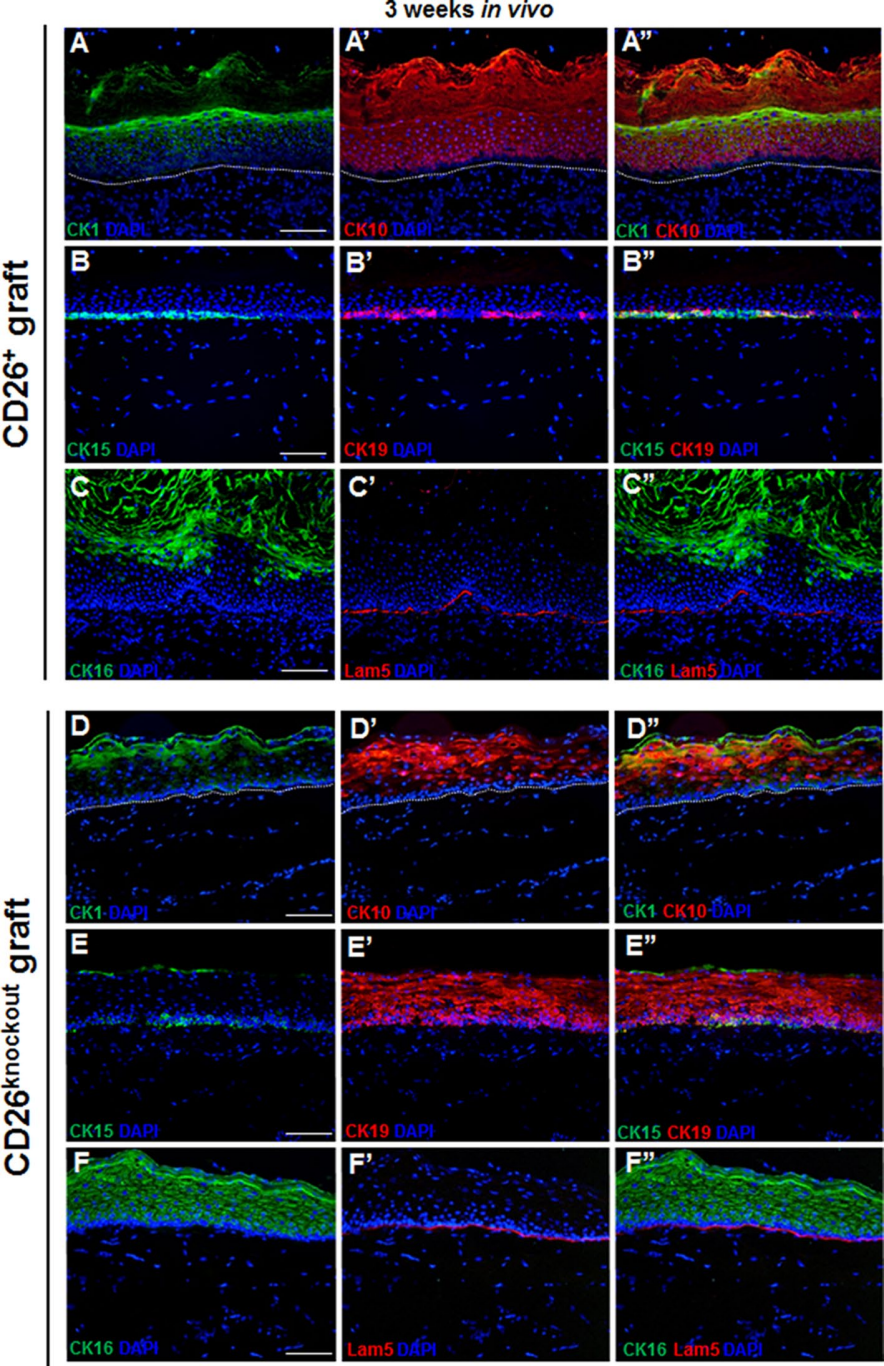
Epidermal stratification and homeostasis in tissue-engineered dermo-epidermal skin grafts 3 weeks after transplantation. (**A–A”** and** D–D”**) Double immunofluorescence stainings using antibodies to CK1 (green) and CK10 (red) in skin grafts containing either (**A**–**A”**) CD26^+^ or (**D**–**D”**) CD26^knockout^ fibroblasts. A delayed stratification process with respect to CK1/CK10 staining is obvious in skin grafts containing (**D**–**D”**) CD26^knockout^ fibroblasts. Dotted lines indicate dermo-epidermal junction. (**B**–**B” and E**–**E”**) Representative immunofluorescence pictures demonstrating the expression of CK15 (green) and CK19 (red) in skin analogs composed of (**B**–**B”**) CD26^+^ or (**E**–**E’**’) CD26^knockout^ fibroblasts. Note a delayed homeostasis in skin grafts containing (**E**–**E”**) CD26^knockout^ fibroblasts. Dotted lines indicate dermo-epidermal junction. (**C–C” **and **F–F”**) CK16 (green) and Lam5 (red) staining demonstrates typical wound healing situation in (**F**–**F”**), whereas in (**C**–**C”**) tissue homeostasis is more advanced. Lam5 indicates dermo-epidermal junction. Blue indicates cell nuclei. Scale bar: 100 µm.

Both CD26^+^ and CD26^knockout^ fibroblasts clearly supported the formation of a stratified epithelium, which consisted of a basal cell layer as well as suprabasal layers (Fig. 6A–F”). However, we observed differences in the homeostasis and morphogenesis of in vivo regenerated epithelium, suggesting that keratinocytes were affected by the level of CD26 expression in the fibroblasts.

To verify in depth keratinocytes differentiation, we immunostained skin substitutes containing either CD26^+^ or CD26^knockout^ fibroblasts for epidermal maturation markers: CK1 and CK10 (Fig. 6A–A”, D–D”). In both types of skin grafts, CK1 expression started in the upper layers of epidermis 3 weeks post-transplantation. However, we observed a delayed stratification process in skin grafts containing CD26^knockout^ fibroblasts with CK10 being expressed only in the uppermost epidermal layers after 3 weeks in vivo (Fig. 6D–D”). On the contrary, CK10 expression in CD26^+^ transplants was visible in all suprabasal epidermal layers as in the native skin (Fig. 6A–A”). The area of CK10 immunofluorescence signal was quantified and presented as a percentage of total area (Suppl. Fig. 3A). The results confirmed the higher expression of CK10 in skin grafts with CD26^+^ fibroblasts suggesting advanced epidermal stratification in these skin substitutes, in comparison to the grafts with CD26^knockout^ cells (75.6 ± 13.9% vs. 16.4 ± 8.5%, respectively; p < 0.001).

We have previously shown, that the homeostatic epithelium is characterized by the basal expression of CK15^21^. CK19, on the other hand, is considered as a marker of young, proliferative human epithelium (detected in the skin of less than 2 years old children). Importantly, the expression of CK19 marker gradually decreases with age and is hardly detected in adult skin^21^. Consistently, we observed CK15 positive cells in the basal cell layer of the epidermis of both types of skin grafts (Fig. 6B–B”, E–E”). CK15/CK19 co-staining, however, revealed that CK19-positive cells were clustered at the basal cell layer and represented a subpopulation of CK15-expressing keratinocytes only in skin graft with CD26^+^ fibroblasts (Fig. 6B–B”). On the contrary, in grafts with CD26^knockout^ fibroblasts, the CK19 expression was visible basally and suprabasally suggesting that tissue homeostasis in this substitute is far from being established (Fig. 6E–E”).

In addition, the significant difference in the expression pattern of the wound healing marker CK16 was observed among both types of skin grafts (Fig. 6C–C”, F–F”). Generally, CK16 is not expressed in normal homeostatic epithelium, however, it is induced in the activated suprabasal keratinocytes in a wound healing situation, including re-epithelization of skin grafts after transplantation^22–24^. We found a strong CK16 staining in all suprabasal epithelial layers of skin substitutes containing CD26^knockout^ fibroblasts, as compared to CD26^+^ skin grafts where the CK16 expression was less pronounced and only visible in the uppermost layers, namely the stratum granulosum and stratum corneum (Fig. 6C–C”, F–F”). Moreover, the quantification of the area of immunofluorescence signals showed an elevated expression of CK16 in skin substitutes containing CD26^knockout^ cells, as compared to grafts with CD26^+^ cells (51.9 ± 8.0 vs. 11.1 ± 6.4%, respectively; p < 0.001) (Suppl. Fig. 3B). This confirms the fact that the period of wound healing is dramatically attenuated in transplants constructed with fibroblasts that entirely lack CD26 expression.

### The influence of CD26^+^ and CD26^knockout^ fibroblasts on the dermal remodeling in vivo

**Figure 7:**
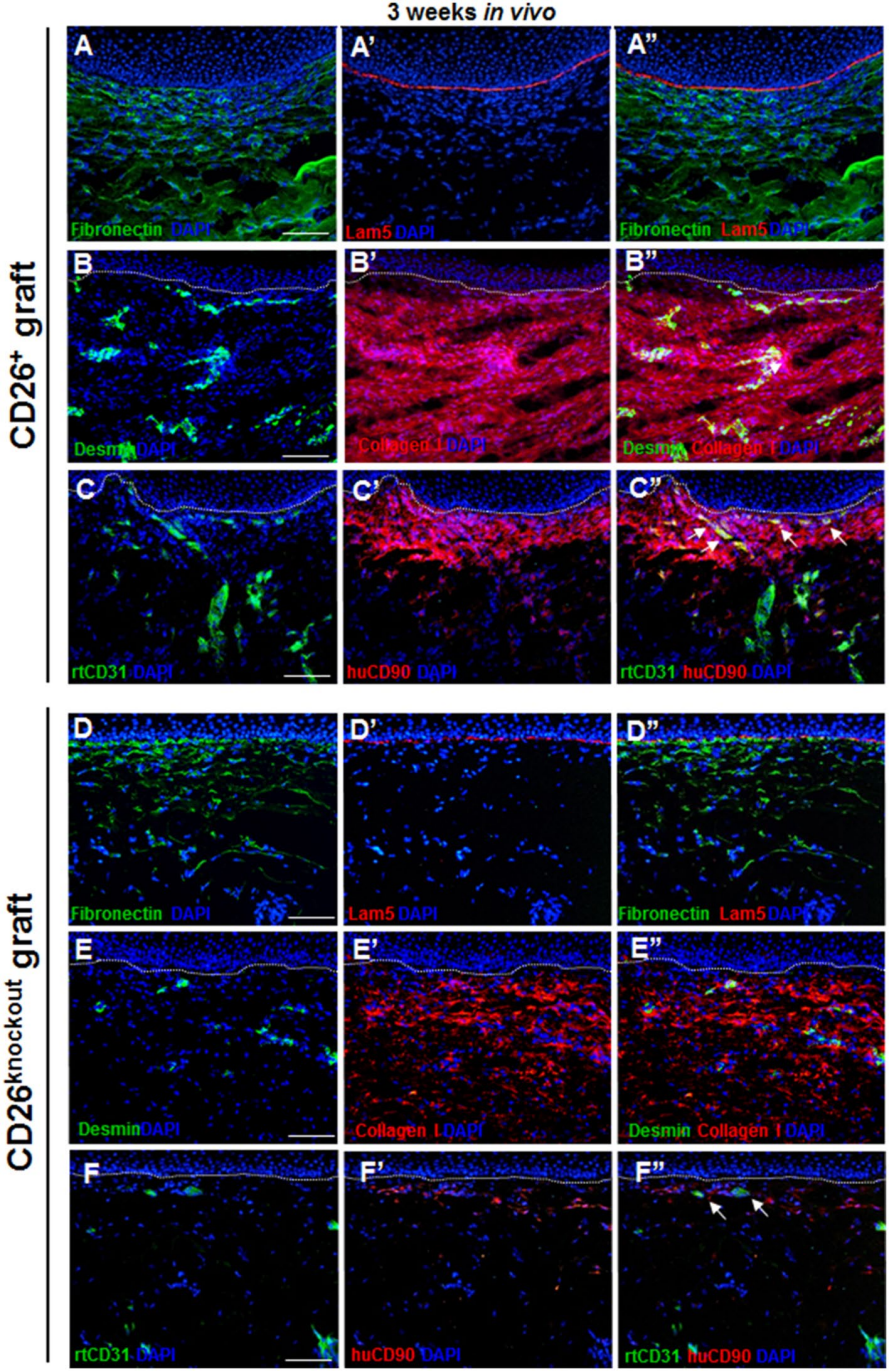
Dermal maturation in tissue engineered dermo-epidermal skin substitutes 3 weeks after transplantation. (**A**–**A”** and** D**–**D”**) Representative immunofluorescence pictures demonstrating the expression of fibronectin (green) and Laminin 5 (Lam5, red) in skin transplants containing (**A**–**A”**) CD26^+^ and (**D**–**D”**) CD26^knockout^ fibroblasts. Fibronectin is distributed throughout dermal compartments up to the dermo-epidermal junction demarcated by Lam5. Note higher expression of fibronectin in skin grafts with CD26^+^ fibroblasts. (**B**–**B”** and** E**–**E”**) Immunofluorescence co-staining of desmin (green) and collagen type I (red) in skin transplants containing (**B**–**B”**) CD26^+^ and (**E**–**E”**) CD26^knockout^ fibroblasts. Please note higher expression of collagen type I in skin grafts with CD26^+^ fibroblasts. (**C**–**C”** and** F**–**F”**). The ingrowth of host CD31-positive blood capillaries (green) into human dermal compartments is highlighted by staining CD90 (red). Note the higher number of rat capillaries (white arrows) in the dermis of skin grafts containing CD26^+^ fibroblasts. Dotted lines indicate dermo-epidermal junction. Blue indicates cell nuclei. Scale bars: 100 µm.

To gain more information about the maturation of dermal compartments of skin grafts containing CD26^+^ and CD26^knockout^ fibroblasts, we performed immunofluorescence stainings for fibronectin, which is an essential component of the dermal extracellular matrix. Interestingly, we observed a significantly higher deposition of fibronectin in skin analogs with CD26^+^ fibroblasts, as compared to the grafts containing CD26^knockout^ cells (Fig. 7A–A”, D–D”). A Laminin-5 co-staining confirmed the presence of the basement membrane in both types of transplants (Fig. 7A–A”, D–D”).

Similarly, we have observed a significantly higher deposition of collagen type I in the dermal compartments of skin grafts with CD26^+^ fibroblasts, as compared to the substitutes containing CD26^knockout^ cells (Fig. 7B–B”, E–E”).

In the following, we investigated the ingrowth of host blood capillaries into human transplants using a specific anti-rat CD31 antibody (Fig. 7C–C”, F–F” and Suppl. Fig. 3C–D”). Transplant with CD26^+^ fibroblasts exhibited a significantly higher number of CD31-positive blood vessels throughout the human dermal compartment demarcated by anti-human CD90 staining (Fig. 7C–C” and Suppl. Fig. 3C–C”). In contrast, the dermis of CD26^knockout^ transplants demonstrated only scarce CD31-positive rat capillaries (Fig. 7F–F” and Suppl. Fig. 3D–D”). In addition, in both transplants, CD31-expressing rat capillaries were decorated with αSMA-positive rat smooth muscle cells (Suppl. Fig. 3C–D”, white arrows).

Subsequently, we sought to investigate if the rapid vascularization and perfusion of CD26^+^ grafts has an effect on epidermal/dermal cell proliferation. To assess this, the expression of Ki67, a marker of cell proliferation, was analyzed in both types of skin analogs (Suppl. Fig. 4A–B”). We showed that indeed the human dermal compartments of CD26^+^ transplants contains significantly more Ki67-positive, hence cycling cells, as compared to the grafts consisting of CD26^knockout^ fibroblasts (Suppl. Fig. 4A’–B’ and C). In accordance to this effect, we also observed a higher density of cells in the human neo-dermis of skin grafts containing CD26^+^ cells (Suppl. Fig. 4A’–B’ and D).

Considering the epidermal compartment, Ki67-expressing cells were almost entirely restricted to the basal epithelial layer of skin graft with CD26^knockout^ fibroblasts, while this marker was visible both basally and suprabasally in CD26^+^ analogs (Suppl. Fig. 4A–B”). Quantification of cycling keratinocytes have revealed that CD26^+^ skin grafts contain 13.7 ± 6.9 Ki67^+^ basal keratinocytes per 1 mm of basal cell layer (Suppl. Fig. 4E). By contrast, 1 mm basal layer of CD26^knockout^ skin grafts consist of 26.9 ± 10.2 proliferating Ki67^+^ keratinocytes. The number of Ki67^+^ suprabasal keratinocytes in CD26^knockout^ skin grafts is 5.5 ± 5.4, while the in the skin substitutes with CD26^+^ fibroblasts this number equals 21.7 ± 4.1 (Suppl. Fig. 4E).

**Figure 8:**
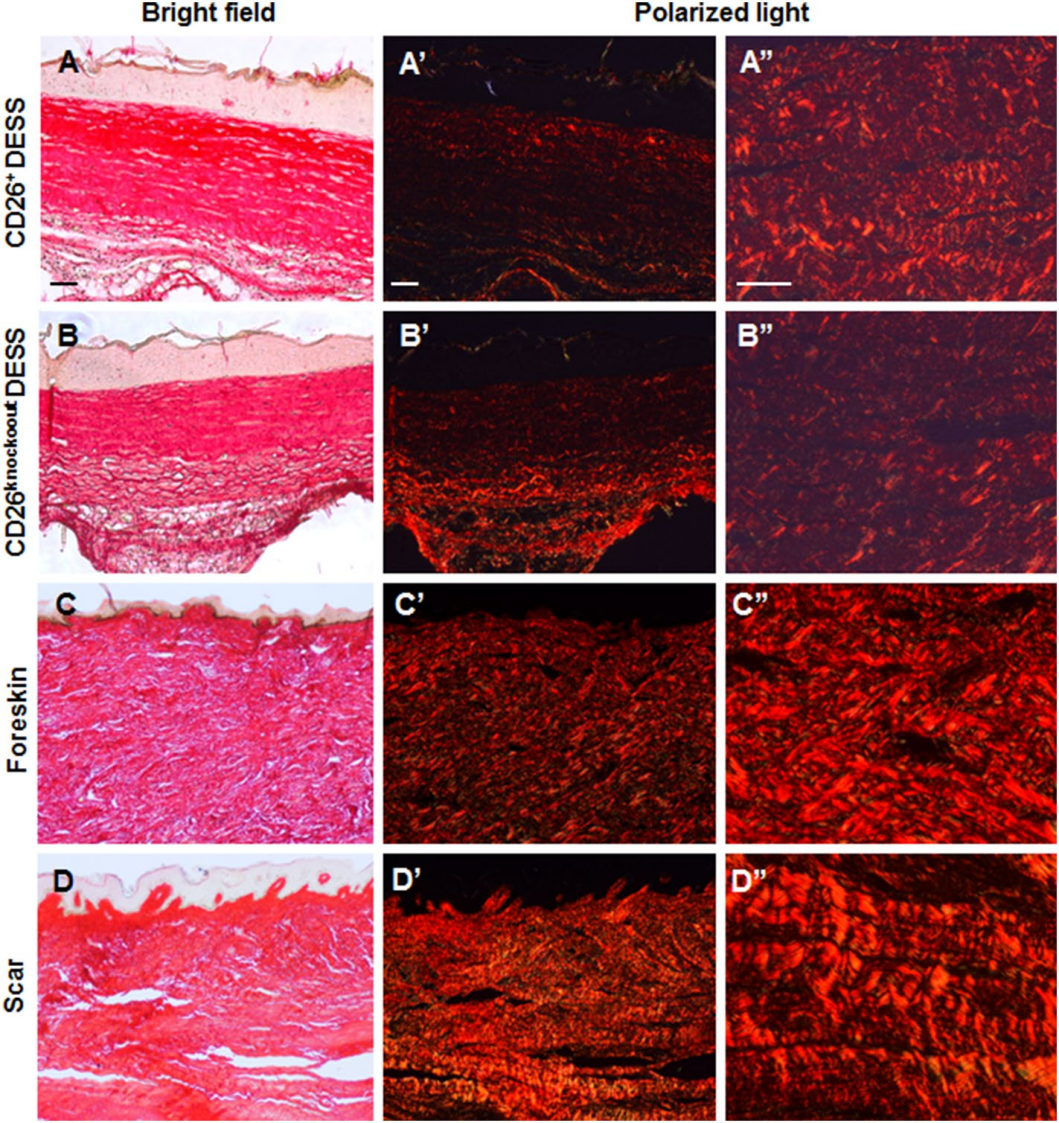
Sirius red staining of CD26^+^ and CD26^knockout^ skin grafts. (**A**–**D”**) Sirius red staining was visualized under bright field (**A**–**D**) as well as under polarized light (**A’**, **A”**, **B’**, **B”**, **C’**, **C”**, **D’**, **D’**’). Pictures in **A”**, **B”**, **C”**, **D”** represent higher magnification of pictures in **A’**, **B’**, **C’**, **D’** respectively. Please note the higher density of collagen fibers in the skin grafts with CD26^+^ fibroblasts (**A”**), as compared to the skin substitutes with CD26^knockout^ cells (**B”**). As a control, Sirius red staining was performed on normal human foreskin (**C**–**C”**) as well as on scar tissue (**D**–**D”**). Please note typical basketweave pattern of collagen fibers in dermis of foreskin as well as parallel collagen fibers in scar tissue. Scale bars: 100 µm.

We have performed Sirius Red staining to assess the collagen content in dermo-epidermal skin substitutes after transplantation (Fig. 8). Sirius red-bound fibrillar collagens were detected under polarized light, where they appear bright red, while the rest of the tissue that remained dark or black. As depicted in Fig. 8, the density of collagen fibers was higher in skin substitutes containing CD26^+^ fibroblasts (A–A”), compared to the skin grafts with CD26^knockout^ cells (B–B”). In addition, Sirius red was performed on normal human foreskin as well as on scar tissue (Fig. 8C–D”). The staining revealed typical basketweave pattern of dermal collagen fibers in normal human foreskin (Fig. 8C–C”). By contrast, in scar tissue collagen fibers were aligned in parallel to each other (Fig. 8D–D”). Compared to the normal human foreskin (Fig. 8C”) and scar samples (Fig. 8D”), we observed a lower amount of collagen in transplanted skin grafts after 3 weeks in vivo (Fig. 8A” and B”). Importantly, the arrangement of collagen fibers was comparable in the transplanted skin grafts (Fig. 8A”,B”) to normal human foreskin samples (Fig. 8C”), but not to scar tissue (Fig. 8D”).

## Discussion

CD26, also known as dipeptidyl peptidase IV, is a multifunctional, membrane-bound protein playing a significant role in the wound healing and scarring processes in the skin of mice^17,25^. However, still little is known about the exact distribution and function of this marker in the human skin. In this study, we analyzed in-depth the expression pattern of CD26 in human skin. We have shown for the first time that expression of this cell surface marker is strongly age-dependent. Furthermore, using our well-established in vivo model, we investigated the impact of CD26^+^ and CD26^knockout^ fibroblasts on the regeneration of tissue-engineered dermo-epidermal skin substitutes (DESS). In particular, we revealed that those two fibroblast subpopulations have a differential effect on the epidermal and dermal homeostasis.

Studies on mesodermal lineage-specific Engrailed-1 (EN1) reporter mice have identified a subpopulation of fibroblasts that is responsible for the ECM deposition during cutaneous wound healing, post-radiation fibrosis and tumor stroma formation^17^. This EN1-expressing subpopulation accounts for approximately 70% of all fibroblasts in adult dorsal skin of mice and can be prospectively enriched by the expression of the cell surface marker CD26^17^. In addition, during mouse skin development, the percentage of EN1-positive (and also CD26-positive) fibroblasts in the dermis continuously increases leading to the phenotypic shift from fetal scar-free regeneration to scar formation in adult skin^26^.

We proved here that the expression of CD26 protein is marginal in fetal and postnatal skin and significantly increases with human age. Similarly to the results in mice presented by Rinkevich^17^, we observed that in the older human dermis (1 and 15 years old foreskin), CD26^+^ population comprises over 80% of total fibroblasts, while in the fetal skin positive cells accounts only for 4%.

Tabib and coworkers used single cell RNA sequencing to identify two major human fibroblast subpopulations, one of which is characterized by the expression of CD26^19^. The pro-fibrotic potential of this population have not been studied in detail in this report, however, a high mRNA expression level of COL1A1 in CD26-expressing cells suggests their possible role in the deposition of ECM^19^. In addition, Vorstandlechner et al. revealed that ECM-associated genes and respective proteins are significantly upregulated in CD26^+^ fibroblasts and suggested that these cells are the main producers of ECM in human skin^27^. Moreover, it has been previously shown that CD26-expressing fibroblasts are selectively increased after injury of human skin and produce the majority of collagen I during ECM remodeling phase of wound healing^28^. In keloids, CD26^+^ fibroblasts exhibited increased proliferative and invasive abilities. This up-regulation of invasive properties of keloid fibroblasts is mediated by CD26 through the IGF-1-induced PI3K/AKT/mTOR signaling pathway. Consequently, inhibition of PI3K pathway leads to the antifibrotic effect in the progression of keloids^29^. In our study, we separated and characterized in detail human CD26^+^ and CD26^-^ fibroblasts in vitro. In accordance to previously published data, our experiments demonstrated that, compared to CD26^-^ population, CD26^+^ fibroblasts are more active in the production of ECM, by means of higher expression of fibronectin as well as higher production and release of cytokine TGFβ1 to the cell culture supernatant in vitro. In addition, the high expression of α-SMA in CD26^+^ fibroblasts suggest that those cells may play a possible role in the production of contractile forces during wound healing^30^.

Vorstandlechner et al., applied magnetic-activated cell sorting (MACS) to separate CD26^-^ and CD26^+^ fibroblasts. The group revealed that initially CD26^-^ cells expressed also low levels of this marker after in vitro expansion^27^. In our experiments, however, the in vitro cultivation of CD26^-^ fibroblasts led to the expression of CD26 in the sorted CD26-negative cells. This phenomenon might be due to 2D cultivation on tissue culture plastic. Furthermore, because of the limited efficiency of FACS, the presence of CD26^+^ fibroblasts in the negative population cannot be excluded.

To overcome the aforementioned limitation of re-gaining the CD26 expression by CD26^-^ fibroblasts in vitro, we used CD26^knockout^ fibroblasts for the preparation of the dermo-epidermal skin substitutes. Interestingly, both CD26^+^ and CD26^knockout^ fibroblasts supported the formation of a stratified, multilayered epithelium with a basement membrane located underneath. However, we observed crucial differences between epithelia of CD26^+^ and CD26^knockout^ transplants. In particular, we found a more pronounced expression of epidermal wound healing marker CK16 in the skin analogs constructed with CD26^knockout^ fibroblasts. Possibly, the epithelium of this skin graft is slower in the establishment of homeostatic state and 3 weeks after transplantation still remains in the wound healing process. Moreover, we observed the persistence of CK19 in all epithelial layers of skin grafts with CD26^knockout^ fibroblasts. We have previously shown that CK19 is a useful marker to evaluate the self-renewing potential of engineered human skin substitutes in vivo^21^*.* This is another line of evidence that epidermal homeostasis in skin grafts consisting CD26^knockout^ fibroblasts is far from being established.

Concerning the dermal compartment, we found a significantly higher expression of fibronectin in skin grafts constructed with CD26^+^ fibroblasts, as compared to the skin analogs with CD26^knockout^ cells. Fibronectin is one of the main components of ECM produced by fibroblasts that play an important role during skin wound healing. Within the granulation tissue, fibronectin organizes into fibrils forming a dense, three-dimensional network that maintains tissue architecture and regulates certain cellular processes, such as cell adhesion^31^, proliferation^32^ and cell migration^33,34^. Our results suggest that CD26^+^ fibroblasts are more active in the production of connective tissue not only in vitro but also in vivo. This, in turn, might be of high importance for cell migration and closure of the skin defects after wounding. Furthermore, we also observed an accelerated ingrowth of rat blood capillaries into the human transplants composed of CD26^+^ fibroblasts in vivo. It is reasonable to mention that the rapid establishment of a functional, hence blood-perfused vascular plexus within the dermis is essential for the adequate nourishment, oxygen supply, and regeneration of the epidermis after transplantation. Of note, we have previously shown that skin substitutes pre-vascularized with adipose-derived stromal cells showed faster achievement of tissue homeostasis and, as a consequence, shorter period of wound healing state^35^. Thereby, our study suggests the advanced dermal maturation of skin constructs composed of CD26^+^ fibroblasts, compared to the analogs with CD26^knockout^ cells.

Our results are also in accordance with previously published data describing the role of CD26-expressing adipose-derived stromal cells (ASC) in the wound healing. The authors revealed that collagen hydrogels seeded with CD26^+^ ASC and used for treating skin burns in mice exhibited accelerated healing as well as improved scar architecture with a more reticular collagen pattern. This is in turn beneficial for the overall tissue architecture and collagen remodeling during wound healing of burn wounds^36^.

Furthermore, we observed increased cell proliferation in the dermal compartment of skin substitutes containing CD26^+^ fibroblasts, hence also higher cell density in the dermis of this skin graft. These findings are in contrast to the study of Pucar et al. who revealed that during the wound healing process, CD26 deficient (CD26^−/−^) mice exhibit increased dermal vascularization as well as higher cell proliferation in both basal epidermal cell layer as well as in dermal compartment of skin^25^. These contradictions may be due to the fact that mouse models, although very successful in studying distinct physiological processes, are also very different from the human skin and a direct comparison is not possible.

In conclusion, this is, to our best knowledge, the first study to demonstrate the effect of primary human CD26^+^ and CD26^knockout^ fibroblasts on the regeneration and maturation of dermo-epidermal skin substitutes in vivo. We have shown here that CD26^+^ fibroblasts are more active in the production of ECM both in vitro and in vivo and are absolutely required to achieve a close to physiological wound healing and a rapid epidermal and dermal homeostasis in vivo. These findings should be taken into account for tissue-engineering of the human skin as different skin biopsies such as split-thickness and full-thickness skin biopsy contain mainly CD26^-^ or a mixture of CD26^+^ and CD26^−^ fibroblasts, respectively. According to our data, also the age of the donor plays a pivotal role in this respect. However, the exact contribution of those two different fibroblasts subpopulations to the wound healing and scarring of human skin still remains to be elucidated.

## Materials and methods

### Human skin samples

All experiments were performed according to the Declaration of Helsinki Principles and after permission by the Ethics Commission of the Canton Zurich (BASEC No. PB_2020_00066 and BASEC Request-No. 2018-00269). Parents or/and patients gave their informed consent. Human foreskin samples were obtained from patients aged between 1 and 15 years and were used for the isolation of keratinocytes and fibroblasts. Human neonatal back skin biopsies were taken from 1 day old donor. Human fetal back skin biopsies were harvested at 24 weeks of gestation and were used for the isolation of fibroblasts. For histological analysis, tissue samples were embedded in OCT (Sakura Finetek, Switzerland) and kept at − 20 °C.

### Isolation and culturing of primary cells

Human fetal fibroblasts were extracted from fetal skin samples, as described previously in^37^. Human postnatal dermal fibroblasts and keratinocytes were isolated and expanded from foreskin samples, as described previously^21,38^. Briefly, skin samples were cut into small pieces and digested overnight at 4 °C in 12 U ml^−1^ dispase (BD Biosciences, Allschwil, Switzerland) in Hank's balanced salt solution containing 5 μg ml^−1^ gentamycin (all from Invitrogen, Basel, Switzerland). Subsequently, epidermis was mechanically separated from dermis using forceps. Epidermis was used for the isolation of keratinocytes while dermis for extraction of fibroblasts. For keratinocytes isolation, epidermal pieces were further digested in 0.5% Trypsin–EDTA (Thermo Fisher Scientific, Basel, Switzerland) at 37 °C for 3 min. Keratinocytes were cultivated in serum free keratinocyte medium (CnT-57, CellnTec, Bern, Switzerland) containing 5 μg/ml gentamycin (Thermo Fisher Scientific, Basel, Switzerland). For fibroblasts isolation, dermis was digested in 2 mg ml^−1^ collagenase blend F (Sigma, Buchs, Switzerland) at 37 °C for ∼ 60 min. Dermal fibroblasts were grown in DMEM supplemented with 10% fetal calf serum (FCS), 4 mM l-alanyl-l-glutamine, 1 mM sodium pyruvate, and 5 μg ml^−1^ gentamycin (all from Thermo Fisher Scientific, Basel, Switzerland).

### Flow cytometry analysis and sorting

Human fibroblasts isolated from fetal skin as well as from neonatal and foreskin samples at different ages were expanded in vitro until passage 1–2. Subsequently, cells were harvested from tissue culture plates using 0.5% Trypsin–EDTA (Thermo Fisher Scientific, Basel, Switzerland). Cell suspensions were stained with CD26-PE antibody (clone M-A261, 1:20, BD Biosciences, Allschwil, Switzerland) and CD90-FITC antibody (clone 5E10, 1:20, Biolegend, Amsterdam, Netherlands) according to manufacturer instructions. Stained cells were either analyzed using BD LSRFortessa flow cytometer (BD Biosciences, Allschwil, Switzerland) or sorted into 2 different groups using BD FACSAriaTM III cell sorter (BD Biosciences, Allschwil, Switzerland): CD26^+^ and CD26^−^ fibroblasts. Both BD LSRFortessa flow cytometer and BD FACSAriaTM III cell sorter were provided by Flow Cytometry Facility at the University of Zurich. For each age, cells were analyzed from 3 independent biological donors.

### Cytospin

Following FACS, separated CD26^+^ and CD26^−^ fibroblasts were incubated for 20 min in suspension with CD26-PE antibody (clone M-A261, 1:20, BD Biosciences, Allschwil, Switzerland). Subsequently, cell suspensions were washed in DBPS, fixed in 4% paraformaldehyde, resuspended in DPBS, and cytocentrifuged using cytofunnels (Thermo Fisher Scientific, Basel, Switzerland) attached to glass slides. Stained cells were visualized using a Nikon Eclipse TE2000-U inverted fluorescent microscope (Nikon, Tokio, Japan).

### Enzyme linked immunosorbent assay (ELISA)

Human/Mouse TGFβ1 ELISA Ready-SET-Go (2nd generation) kit (eBioscience, Vienna, Austria) was used according to manufacturer’s instructions to measure the concentrations of TGFβ1 in the conditioned media of CD26^+^ and CD26^−^ fibroblasts isolated from foreskin samples. Briefly, CD26^+^ and CD26^−^ fibroblasts were cultured in standard fibroblast medium supplemented with 10% FCS, HEPES and penicillin/streptomycin at 70% confluence. Conditioned media of CD26^+^ and CD26^-^ fibroblasts were collected after 72 h of incubation, centrifuged at 300×*g* for 5 min, and filtered through a 0.22-μm syringe filter. To activate the latent TGFβ1 to immuno-reactive form, samples were treated with 1 N HCl and thereafter neutralized with 1 N NaOH. TGFβ1 capture antibody was used to coat Corning Costar 9018 ELISA plate (100 µl of antibody/well). The plate was incubated with antibody overnight at 4 °C. On the following day, solution was aspirated, and the wells were washed three times with 250 µl of washing buffer. Subsequently, the wells were blocked with 200 µl of 1 × ELISA/ELISPOT for 1 h at room temperature. 100 µl of standard or samples were added to appropriate wells, incubated overnight at 4 °C and washed three times with 250 µl of washing buffer on the next day. Avidin-HRP was added to the respective wells and incubated for 30 min at room temperature. Wells were then washed 5 times, and treated with 1 × TMB Solution (100 µl/well) for 15 min at room temperature. To stop the reaction, the stop solution was added into each well (50 µl/well) and the plates were analyzed at 450 nm using a spectrophotometer. To determine the baseline concentrations of TGFβ1, culture media were used. All experiments were run in triplicate using conditioned media from three independent biological donors.

### Western blotting

Human CD26^+^ and CD26^-^ fibroblasts were cultivated in vitro until they reached 80% confluency. Subsequently, cells were lysed in RIPA buffer containing protease inhibitor cocktail (BioRad, Cressier, Switzerland) for 5 min on ice. Cell scrapers were used to collect the lysates, and solutions were centrifuged at 14,000×*g* for 15 min at 4 °C. Samples were mixed with Laemmli Buffer and loaded to the wells of pre-casted gels (BioRad, Cressier, Switzerland). Electrophoresis was run in 1 × Running Buffer (BioRad, Cressier, Switzerland) for about 35 min. Thereafter, proteins were transferred from the gel to the nitrocellulose membrane using Trans-Blot Turbo transfer system (BioRad, Cressier, Switzerland). Immediately after protein transfer, the membrane was incubated with blocking solution containing 5% BSA in TBST for 60 min at room temperature on a shaker. The αSMA (clone 1A4, 1:100, Baar, Switzerland) primary antibody was added to the membrane and incubated overnight at 4 °C on a shaker. On the next day, the membrane was washed five times in 1 × TBST at room temperature. The membrane was incubated with horseradish peroxidase (HRP)-labeled anti-mouse secondary antibody for 1 h at room temperature. Subsequently, the membrane was again washed extensively with 1 × TBST and incubated with substrate solution (BioRad, Cressier, Switzerland) for 3–5 min. The results were visualized using Syngene G-Box (Syngene, United Kingdom).

### CD26 knockout using lenti-CRISPR/Cas9 viral vectors

A lentiviral gene transfer system was applied to inhibit the expression of CD26 in human primary skin fibroblasts. The 20-nucleotide gRNA sequence (GTTGTGAGCTGAATCCGGAA) was obtained from http://crispr.mit.edu/. A lentivirus CRISPR vector for Cas9 and the gRNA co-expression was designed in VectorBuilder (VectorBuilder Inc., Chicago, IL). The plasmid sequence is shown in the Supporting Information.

Lentiviral vectors were produced by transient transfection of HEK293FT cells. The cells were co-transfected with the transfer vector and VSVG, REV, and MDL plasmids using the calcium phosphate method at a plasmid molar ratio 1:1:1:1. A total of 1 × 10^6^ cells were seeded in 25 cm^2^ tissue culture flasks 24 h before transfection. Cells were refed with fresh complete medium 2 h before transfection. The final transfection mixture had a total DNA amount of 10 µg and a CaCl_2_ concentration of 125 mM. Fresh complete medium (4 ml) was added to the cells 20 h post-transfection. Twenty-four hours later, the viral supernatant was collected, filtered through a 0.45 µm syringe filter and concentrated to a final volume of 0.05 mL (approximately 80-fold) using Amicon® Ultra Centrifugal Filter Units 100 KDa (Merck Millipore, Darmstadt, Germany). The aliquots were stored at − 80 °C.

Human primary skin fibroblasts were grown to 80% confluency in 24-well plates and transduced with 10 µl lentiviral concentrate complexed with 5 µg Polybrene (Sigma-Aldrich) in 0.5 ml complete medium. The next day, the cells were washed, and fresh medium was added.

At 48 h post-infection, knockout cells were selected with blasticidin. The CD26 knockout was confirmed by immunostaining on live cells with the antibody (clone M-A261, 1:20, BD Biosciences (Allschwil, Switzerland) followed by flow cytometry analysis using a FACSAriaIII equipped with the FACSDiva® Software (BD Biosciences, Allschwil, Switzerland).

### Preparation of dermo-epidermal skin analogs

To prepare dermo-epidermal skin substitutes, 1 × 10^5^ of stromal cells (CD26^+^ or CD26^knockout^) were mixed with collagen type I (Symatese, France) and casted into 6-well cell culture inserts (3.0 µm pore-size membranes) (BD Falcon, Switzerland). These dermal compartments were cultivated in vitro for additional 7 days in DMEM medium supplemented with 10% fetal calf serum (Invitrogen, Switzerland), 5% Pen/Strep, 5% HEPES. Thereafter, 8 × 10^5^ of keratinocytes from interfollicular epidermis were seeded onto the dermal equivalents. Dermo-epidermal skin substitutes were cultivated in vitro for additional 5 days and subsequently they were transplanted onto back of immune-incompetent rats.

### Transplantation of cultured skin substitutes

The experimental procedures were approved by the Local Committee for Experimental Animal Research (Cantonal Veterinary Office Zurich, permission number: ZH090/2015) and performed in accordance with relevant guidelines and regulations. We confirm that the study was reported in accordance with ARRIVE guidelines. Eight to ten weeks old female Nu/Nu rats (Charles River, Freiburg, Germany) were anesthetized by inhalation of 5% Isofluran (Baxter, Volketswil, Switzerland), and maintained by inhalation of 2.5% Isofluran via mask as described previously in^39,40^. The dermo-epidermal skin substitutes were transplanted on full-thickness skin wounds created on the back of the rats. Custom made surgical steel rings (diameter 2.6 cm) were sutured to the skin of rats, to prevent from wound closure. As a wound dressing, Silicon foil (Silon-SES, BMS, USA), a polyurethane sponge (Ligasano, Ligamed, Austria) and tape (Leukoplast, BSN medical, Germany) were applied. Dressing changes were made once per week. The animals were euthanized by CO_2_ three weeks after transplantation and skin grafts were excised and embedded in OCT compound for further analysis.

### Immunohistochemical staining and analysis

10–12 µm cryosections or cells on cell culture dishes were fixed in acetone/methanol 1:1 mixture for 5 min at − 20 °C. Subsequently, slides or plates were blocked with 2% Bovine Serum Albumin in PBS and incubated with primary antibodies either overnight at 4 °C or 1 h at room temperature. On the following day, slides or plates were washed 3 times in PBS and incubated with corresponding FITC or TRITC-conjugated secondary antibodies for 45 min at room temperature. To stain cell nuclei, 4′,6-Diamidin-2-phenylindol (DAPI) was used. Finally, after additional washing in PBS, slides were mounted in mounting medium (Sigma-Aldrich, Buchs, Switzerland) and cover-slipped. To take pictures of immunofluorescence stainings, a Nikon Eclipse TE2000-U inverted microscope equipped with Hoechst, FITC and TRITC filter sets (Nikon AG, Egg, Switzerland; Software: Nikon ACT-1 vers. 2.70) was used. The images were taken by a connected digital camera (DXM1200F) and were further processed with Photoshop 11.0.

For immunofluorescence analysis the following antibodies were used: CD26-PE (clone M-A261, 1:20) from BD Biosciences (Allschwil, Switzerland); CD90-FITC (clone 5E10, 1:20) from Biolegend (Amsterdam, Netherlands); Ki67 (clone B56, 1:200), CD31 (clone TLD-3A12, 1:100) all form BD Pharmingen (Basel, Switzerland); fibronectin (polyclonal, 1:100) from Abcam (Germany); αSMA (clone 1A4, 1:100), CK19 (clone RCK108, 1:50), CK10 (clone DE-K10, 1:100) all from Dako (Baar, Switzerland); CD90 (clone AS02, 1:100) from Merck Millipore (Darmstadt, Germany); TGFβ1 (clone 9016, 1:100) from R&D Systems (Germany); CK15 (clone SP190, 1:100) from Santa Cruz (Heidelberg, Germany); CK1 (clone LHK1, 1:200) from Progen (Heidelberg, Germany); Laminin-5 (clone P3H9-2, 1:100) from Labforce (Nunningen, Switzerland); CK16 (clone LL025, 1:50) from Invitrogen (Basel, Switzerland).

### Quantification of the area of TGFβ1, αSMA, fibronectin and collagen immunofluorescence staining

For each sample, five sections (20 × magnifications) were capture to quantify the area of TGFβ1, αSMA, fibronectin and collagen immunofluorescence signals (n = 5 per condition; from 3 independent donors/experiments). The positively stained area was measured using the same macroscopic settings and quantified using NIH ImageJ software. The positively stained area was normalized to the number of cells. P value was calculated using unpaired student t-test.

### Quantification of the area of CK10 and CK16 immunofluorescence staining

For each sample, five sections (20 × magnifications) were captured to quantify the area of CK10 and CK16 immunofluorescence signals (n = 5 per condition; from 3 independent donors/experiments). From each picture, the region of interest was chosen. The positively stained area was measured in the region of interest and is presented as percentage of total area of region of interests. The quantification was performed using NIH ImageJ software. All pictures were taken using the same macroscopic settings.

### Quantification of Ki67^+^ cells in the epidermal and dermal compartments of transplanted skin grafts

Transplanted skin grafts were stained for Ki67. In order to examine the proliferation of fibroblasts, the specific regions of interest was chosen in the dermal compartments of transplanted skin grafts. Ki67^+^ fibroblasts were calculated in the regions of interest and results are presented as the number of proliferating Ki67^+^ fibroblasts per 1 cm^2^ of dermis. In order to examine proliferating keratinocytes, Ki67^+^ cells located on the epidermal basal cell layer were quantify. Results are presented as the number of proliferating Ki67^+^ keratinocytes per 1 mm of basal cell layer. To verify the proliferation of keratinocytes in the epidermal suprabasal cell layers, the respective regions of interest were chosen. The Ki67^+^ cells were quantified in the regions of interest. The results are presented as the number of Ki67^+^, proliferating keratinocytes per 1 cm^2^ of epidermis. Five different regions of interest were chosen for each group (n = 5).

### Quantification of the density of cells in the dermal compartments of transplanted skin grafts

In order to quantify the total density of fibroblasts in transplanted skin grafts, the regions of interest were chosen in the dermal compartments. The total number nuclei (stained with DAPI) was quantified using NIH ImageJ software. Results are presented as the number of cells per 1 cm^2^ of dermis. Five different regions of interest were chosen for each group (n = 5).

### Statistical analysis

All results are reported as mean ± standard deviation (SD). GraphPad Prism 4.0 (Graph Pad software, La Jolla, CA, USA) was used to perform the statistical analysis. The unpaired Student’s t-test was used to perform the comparison between two groups. Results were considered significant according to GraphPad Prism: *indicates p-value 0.01 to 0.05 (significant), **Indicates p-value 0.001 to 0.01 (very significant), ***Indicates p < 0.001 (extremely significant), ns = not significant (p > 0.05).

## Supplementary Information

Supplementary Information.
