# Supplementary material for: The influence of CD26+ and CD26− fibroblasts on the regeneration of human dermo-epidermal skin substitutes
Source: Sci Rep. 2022 Feb 4;12:1944. doi: 10.1038/s41598-022-05309-5 (PMC8816920; doi:10.1038/s41598-022-05309-5)
Supplement: Supplementary file 1 — Supplementary Information. [file 41598_2022_5309_MOESM1_ESM.docx]

**The influence of CD26^+^ and CD26^-^ fibroblasts on the regeneration of human dermo-epidermal skin substitutes**

Katarzyna Micka^1,2^, Agnes S. Klar^1,2^, Athanasia Dasargyri^1,2^, Thomas Biedermann^1,2^, Ernst Reichmann^1,2^, Ueli Moehrlen^1,3,4 *^

^1^Tissue Biology Research Unit, Department of Surgery, University Children’s Hospital Zurich, University of Zurich, Switzerland

^2^Children’s Research Center, University Children’s Hospital Zurich, Zurich, Switzerland

^3^Department of Surgery, University Children’s Hospital Zurich, University of Zurich, Switzerland

^4^University of Zurich, Zurich, Switzerland

*Corresponding author:

Ueli Moehrlen

University Children’s Hospital Zurich

Department of Surgery

Steinwiesstrasse 75

8032 Zurich

Switzerland

Email: [Ueli.Moehrlen@kispi.uzh.ch](mailto:Ueli.Moehrlen@kispi.uzh.ch)

**Supplementary figures**

**
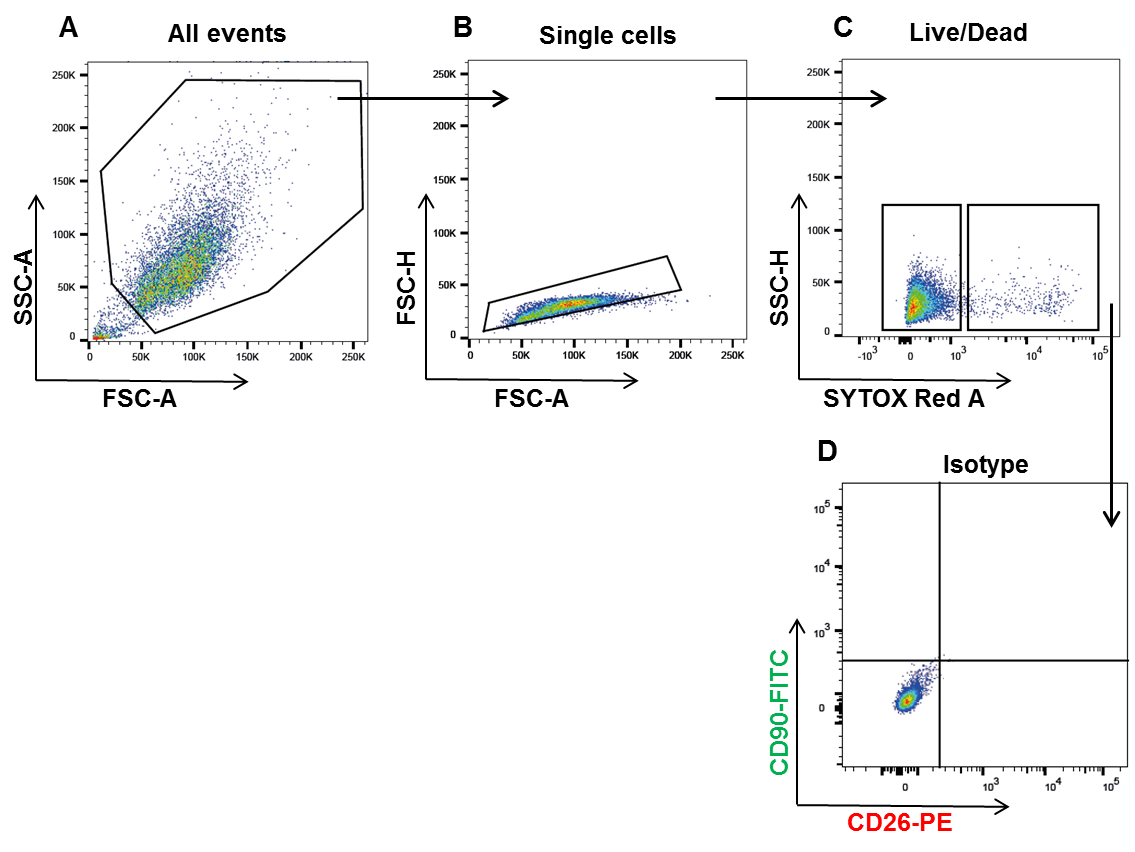
**

**Supplementary Figure 1. Gating strategy used during fluorescence-activated cell sorting (FACS). (A-D)** During FACS, gating strategy involves the exclusion of cell doublets (A and B) as well as the elimination of dead cells following SYTOX Red A staining (C). Isotype control was used to identify background signal and correctly set the gates for positive cells (D).

**
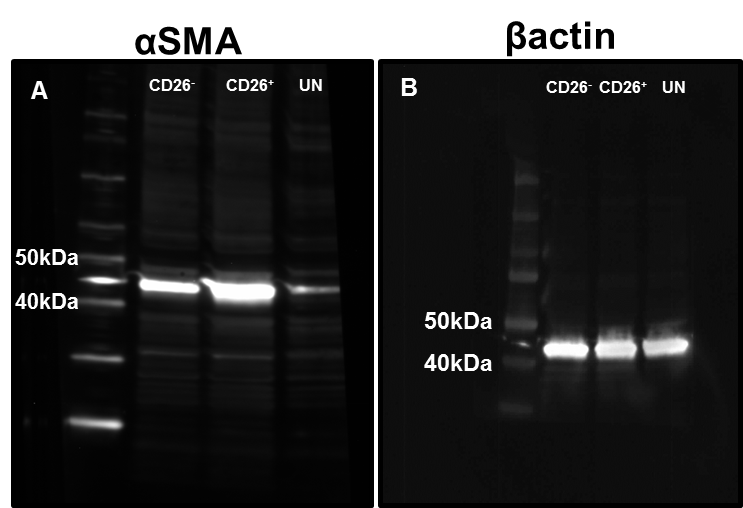
**

**Supplementary figure 2. Raw data of western blot analysis. (A-B)** Western blot analysis of αSMA protein isolated from cell lysates of cultured CD26^+^, CD26^-^ and unfractioned (UN) fibroblasts. Please note the higher expression of αSMA in CD26^+^ fibroblasts, in comparison to the CD26^-^ cells. βactin was used as an internal control.

**
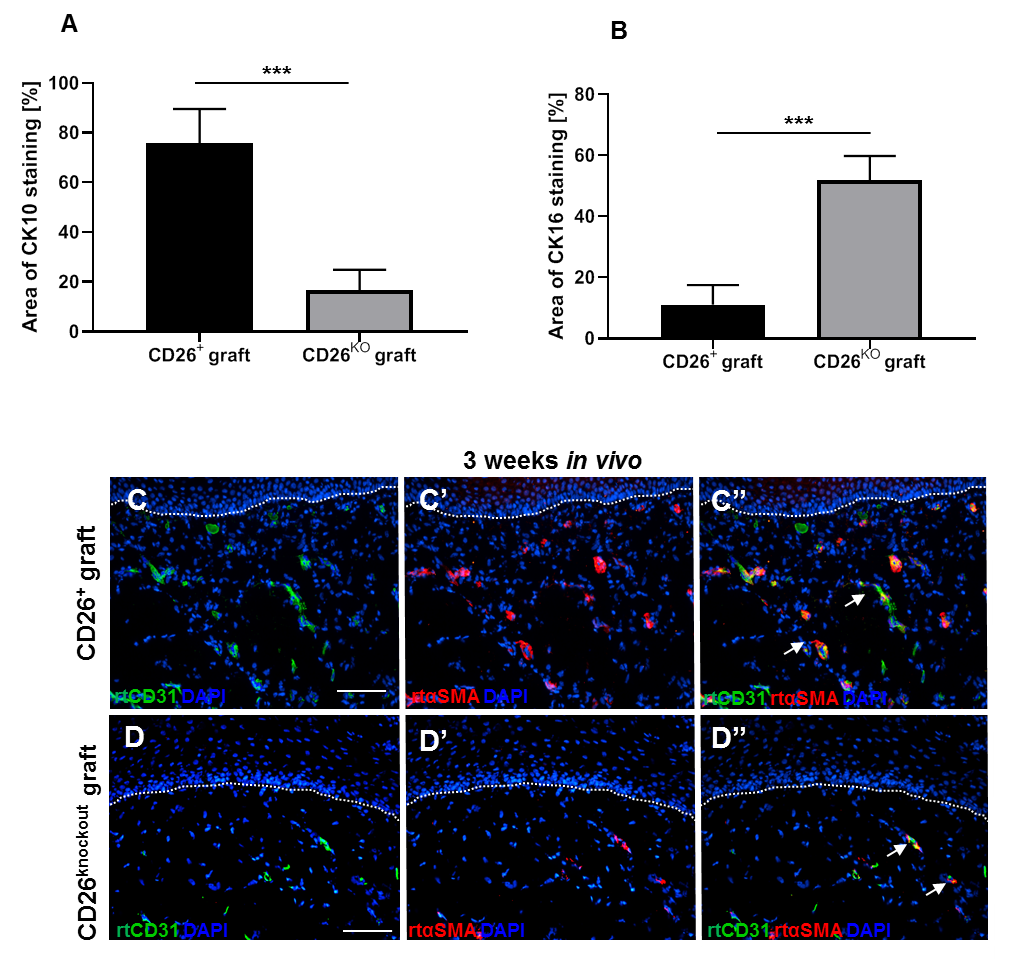
**

**Supplementary Figure 3. Quantification of immunofluorescence signal as well as the ingrowth of host rat blood capillaries into human skin grafts *in vivo*.** **(A-B)** Quantification of the area of (A) CK10 and (B) CK16 immunofluorescence staining. Please note the higher expression of CK10 (75.6 ± 13.9% vs. 16.4 ± 8.5%) and lower expression of CK16 (11.1 ± 6.4% vs. 51.9 ± 8.0) in skin substitutes with CD26^+^ fibroblasts, as compared to the grafts with CD26^knockout^ cells. Data are presented as a mean ± SD (n=5). P-values were calculated using unpaired student t-test, *** indicates P-value p<0.001 (extremely significant). (**C-D’’**) Rat CD31 positive (green) lumenized capillaries are surrounded by rat αSMA^+^ smooth muscle cells (red, white arrows). Note also the higher number of CD31^+^ rat capillaries in the dermis of skin grafts containing CD26^+^ fibroblasts. Blue indicates cell nuclei. Scale bars: 100µm.


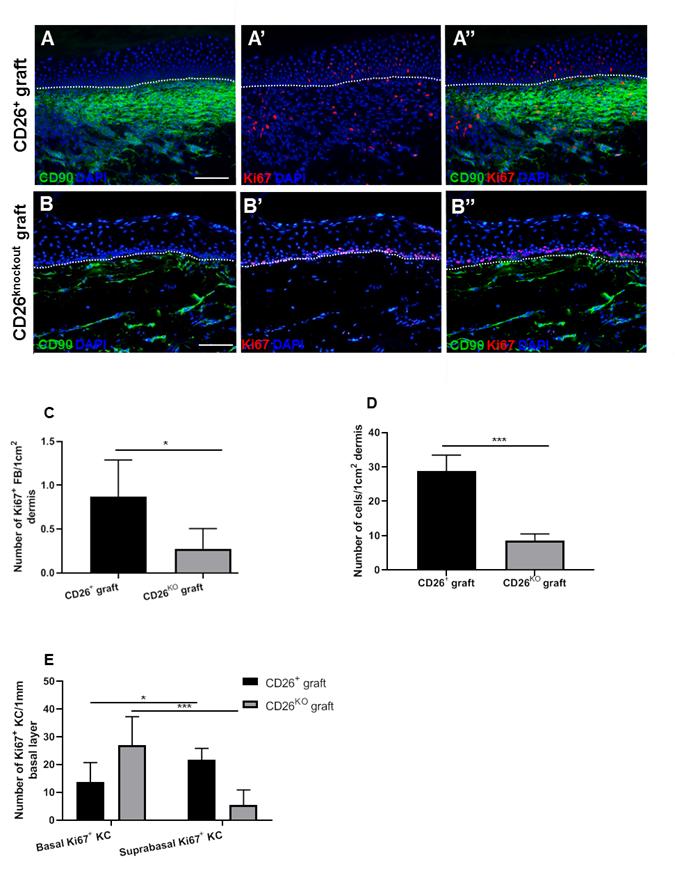


**Supplementary Figure 4. Proliferation of fibroblasts and keratinocytes in transplanted CD26^+^ and CD26^knockout^ skin grafts.** (**A-B’’**) Representative immunofluorescence pictures showing the presence of proliferating, Ki67^+^ keratinocytes in epidermal compartments as well as Ki67^+^ fibroblasts in dermal compartments of transplanted skin grafts containing CD26^+^ or CD26^knockout^ cells (**C**) Quantification of cycling, Ki67^+^ fibroblasts in the dermal compartments of CD26^+^ and CD26^knockout^ skin grafts. Please note that dermal compartment of skin substitutes with CD26^+^ fibroblasts contain higher number of Ki67^+^ proliferating fibroblasts than the skin grafts with CD26^knockout^ cells. Data are presented as a mean ± SD (n=5). P-values were calculated using unpaired student t-test. * indicates P-value 0.01 to 0.05 (significant). (**D**) The overall quantification of the number of cells in the dermal compartments of CD26^+^ and CD26^knockout^ skin substitutes. Please note the higher density of cells in dermis of CD26^+^ skin grafts. Data are presented as a mean ± SD (n=5). P-values were calculated using unpaired student t-test. *** indicates P-value p<0.001 (extremely significant). Blue indicates cell nuclei. Scale bars: 100µm. (**E**) Quantification of proliferating cells revealed that CD26^+^ skin substitutes contain significantly more proliferating, Ki67^+^ keratinocytes in epidermal suprabasal layers, in comparison to the skin grafts with CD26^knockout^ cells. By contrast, the number of cycling keratinocytes in basal cell layer of CD26^+^ skin grafts is significantly reduced, as compared to the skin substitutes with CD26^knockout^ cells. Data are presented as a mean ± SD (n=5). P-values were calculated using unpaired student t-test. * indicates P-value 0.01 to 0.05 (significant); *** indicates P-value p<0.001 (extremely significant).
